# Room to Breathe: The Impact of Oxygen Rationing on Health Outcomes in SARS-CoV2

**DOI:** 10.3389/fmed.2020.573037

**Published:** 2021-01-06

**Authors:** Daniel K. Goyal, Fatma Mansab, Sohail Bhatti

**Affiliations:** ^1^COVID-19 Research Group, Public Health Gibraltar, Gibraltar Health Authority, Gibraltar, United Kingdom; ^2^Department of Medicine, Acute General Medicine, St. Bernard's Hospital, Gibraltar, United Kingdom; ^3^Department of Postgraduate Medicine, University of Gibraltar, Gibraltar, United Kingdom; ^4^Director of Public Health, Gibraltar Health Authority, Gibraltar, United Kingdom

**Keywords:** guidelines, hypoxia, SARS - CoV-2, treatment, oxygen, COVID-19, healthcare, rationing

## Abstract

As the primary surge of coronavirus disease 2019 (COVID-19) wanes in many countries, it is important to reconsider best practice. More cases, probably the majority of cases, are yet to come. Hopefully, during this next phase, we will have more time, more resources, and more experience from which to affect better outcomes. Here, we examine the compromised oxygen strategy that many nations followed. We explore the evidence related to such strategies and discuss the potential mortality impact of delaying oxygen treatment in COVID-19 pneumonia.

## Introduction

Severe acute respiratory syndrome coronavirus 2 (SARS-CoV2) is a new viral infection in humans, causing what has been termed COVID-19 (coronavirus disease 2019). For many, the illness is mild, often causing an upper respiratory tract infection (URTI). For a few, the disease progresses to a lower respiratory tract infection, and invariably, chest imaging shows this as a viral pneumonia (1). Again, most recover, but for some, the disease does not abate, and they go on to develop acute respiratory distress syndrome (ARDS) (2).

The rate of progression from mild (URTI or other viral constellation) to severe (pneumonia) to critical (ARDS) is not entirely clear. Estimates range between 76 and 99% for mild disease, 1 and 24% for progressive pneumonic illness, and 0.6 and 13% for further progression to ARDS. The true mortality rate also remains relatively unclear with estimates of <1% to over 10% and is likely to depend in part on access to appropriate health care (3–5).

Wuhan (China), Lombardy (Italy), Madrid (Spain), London (UK), and New York (USA) each experienced a surge of cases to the point where the local health services struggled to provide optimal care to all patients (6). During this “surge” period, the decision was made by some healthcare systems to ration oxygen, i.e., to delay the initiation of oxygen and to permit patients to maintain lower oxygen levels than would normally be accepted (7).

Such “conservative” oxygen strategies continue in many parts of the world despite adequate healthcare capacity and resources (8–12). The concern then is that patients are going without optimal treatment not due to excess demand on the health service but due to a practice established during actual or feared resource limitations.

To appreciate the impact of oxygen rationing on health outcomes during the COVID-19 pandemic, the evidence behind the “conservative” oxygen approach was examined, specifically for mortality outcomes.

## The Guidelines

The normal, mean oxygen saturations (SpO2) across an adult population has been reported as 97.5% (±1.5%) within a mean age of 63 years (range, 38–83 years). Approximately 7% of the population is classified as hypoxic at baseline (SpO2 <95%) (13).

Current treatment guidelines for community acquired pneumonia, including viral pneumonia, recommend commencement of supplemental oxygen when SpO2 falls below 95% and recommend a maintenance target SpO2 of 94–98% in the majority of patients (14).

The recent World Health Organization (WHO) COVID-19 treatment guidelines recommend to maintain target SpO2 of >90% in adults and between 92 and 95% in pregnant women (15).

The COVID-19 guidelines produced by the Surviving Sepsis Campaign (SSC) suggest (at recommendation 23) that supplemental oxygen should be commenced when a patient's SpO2 is <92%. The guidelines also recommend a “conservative oxygen” strategy for all patients with COVID-19 aiming for target SpO2 between 92 and 96%. The panel go on to explain how their concern for potential resource limitations influenced the clinical recommendations (16):

“Considering the associated patient harm at the extremes of SpO2 targets and the increased cost of liberal oxygen use, as well as the potential to reduce equity if oxygen resources are depleted, the panel issued a strong recommendation against using oxygen to target SpO2 >96%, and a strong recommendation to avoid lower values (SpO2 <90%)” (16).

## The Evidence Behind The “Conservative Oxygen” Strategy

The WHO references its own general pediatric guidelines. The SSC guidelines reference the five most pertinent publications, reviewed below (17–21).

The first was a retrospective analysis published in March 2020, investigating the optimal oxygen saturations (SpO2) for patients admitted to Intensive Care Units (ICU). The investigators analyzed over 35,000 patients and concluded that the optimum oxygen range for patients in ICUs was 94–98%. Patients who spent only 40% in the optimal range had a 50% increased mortality than those who spent 80% in the optimal range of SpO2 94–98% (17). The investigators corrected for a number of confounders, including disease severity.

The second study referenced by the SSC guidelines is the Improving Oxygen Therapy in Acute Illness (IOTA) meta-analysis examining liberal vs. conservative oxygen use in patients with a variety of conditions [stroke (*n* = 8), myocardial infarction (*n* = 6), cardiac arrest (*n* = 2), acute appendicitis (*n* = 2), critical care (*n* = 2), sepsis (*n* = 1), septic shock (*n* = 1), perforated viscus (*n* = 1), limb ischemia (*n* = 1), and traumatic brain injury (*n* = 1)] (18).

None of the studies reviewed in the IOTA meta-analysis examined pneumonia. Perhaps of some relevance are the two critical care studies analyzed (22, 23).

The first was a randomized controlled trial (RCT) examining the effect of conservative oxygen (defined in this study as SpO2 of 94–98%) and liberal oxygen (97–100%) on mortality in an ICU. The supplementary data suggest that the liberal group was significantly more acutely unwell *on admission* than the conservative group (for example, respiratory failure of 30.2 vs. 17.4%; mechanical ventilation at admission of 27 vs. 16.1%; liver failure of 33.3 vs. 22.5%; and renal failure of 45.7 vs. 25%). This RCT reported improved mortality in the conservative, SpO2 94–98% group (22).

The second critical care study examined in the IOTA meta-analysis was a pilot RCT study examining conservative oxygen targets (88–92%) vs. liberal oxygen targets (>96%) in patients on mechanical ventilation (*n* = 103). It is of note that 21% of the conservative arm were chronic obstructive pulmonary disease (COPD) patients vs. only 10% of patients in the liberal oxygen arm. The mean oxygen levels in each group over the study period were 93.4% in the conservative arm and 97% in the liberal arm. The study did not identify any difference in mortality between the two groups (23).

All of the other trials analyzed in the IOTA meta-analysis had little relevance to optimum oxygen saturations in patients with pneumonia or ARDS. Most of the studies were testing supplemental oxygen as a potential treatment (for example in stroke or myocardial infarction). Further, patients who were hypoxic were excluded from almost half the trials analyzed. The mean oxygen saturation (where the data was available) in the liberal group was 96.4%, and in the conservative group was 96.7%. Following trial sequential analysis, the authors concluded that hyperoxemia carried an increased 1-year mortality risk [hazard ratio (HR), 1.11 (95% CI 1.00–1.24), *p* = 0.05]. The authors go on to suggest that the ideal target SpO2 for all admissions *might* be 94–96%. With the regression analysis likely powered by the true hyperoxemic trials and the marked heterogeneity within the studies analyzed, the reason for the authors equivocation seems justified (18).

The third paper referenced in the SSC guidelines was an opinion paper making recommendations based on the IOTA meta-analysis (19).

The fourth paper was an RCT examining conservative (90–96%) vs. normal oxygen targets during mechanical ventilation in the ICU (*n* = 1,000). Over 22 days, the conservative group spent 28 h more with an Fio2 of <0.21 than the usual group, and a total of 22 h less time above SpO2 of 96% than the usual group. The mean oxygen level range over the course of the study were PaO2 of 80–85 mmHg (~SpO2 of 95–96%) in the conservative group and PaO2 of 90–95 mmHg (~SpO2 of 96–97%) in the normal group. There was no significant mortality difference noted (20).

The final paper referenced was an RCT into the use of conservative oxygen levels (sats 88–92%) vs. liberal oxygen use (sats >96%) in ARDS (*n* = 205). Relatively strict adherence to target saturations were observed with a mean range of SpO2 of 92–93% in the conservative group vs. 95–97% in the liberal group. The study was halted due to excessive death in the conservative oxygen arm vs. the liberal arm (44.4 vs. 30.4% 90-day mortality). As well as the 23% increased ICU mortality, 27% increased 28-day mortality, and the 50% higher 90-day mortality, the complications in the conservative arm included mesenteric ischemia (5 vs. 0%) and cardiac arrhythmias (44 vs. 28%). Target oxygen saturations lower than that currently recommended were shown to substantially increase mortality (21).

## Discussion

Instigating a “conservative oxygen” strategy as a means of healthcare rationing is likely to contribute to the higher mortality experienced during a COVID-19 “surge.” The evidence for a significant increase to mortality rate was particular strong for targeting lower oxygen saturations (<94%). The evidence of harm when restricting the upper limit of oxygen saturations (98–96%) was less convincing (Table 1). It is of note that the recent British Thoracic Society (BTS) guidelines specifically for COVID-19 advises a target SpO2 of 94–98% (25).

**Table 1:** Relevant studies comparing conservative vs. liberal oxygen strategies in acute illness.

**Study**	**Methods**	**Findings**	**Comment**	**Suggestions**
Chu et al. (18)	Meta-analysis (up to Oct 2017) of trials comparing liberal vs. conservative oxygen strategies in a variety of conditions(*n* = 16,037)	“hyperoxemia” carries an increased 1-year mortality risk [HR 1.11 (95% CI 1.00–1.24), *p* = 0.05)	Heterogenous studies, examining oxygen therapy mainly in ischemic conditions. Unreliable in relation to oxygen targets in acute nonischemic illness.	Optimum target saturations *might* become unfavorable above SpO2 of 94–96%
Girardis et al. (22)	Single-centered, open-labeled RCT Conservative (SpO2 of 94–95%) vs. liberal (SPO2 of 97–100%) in ICU admissions of any cause (*n* = 434)	Improved mortality in conservative group (SpO2 of 94–98%): RR 0.57 (95% CI 0.37–0.9, *p* = 0.01)	Liberal group had significantly more medical problems at enrolment.	Target sats of 94–98%
Panwar et al. (23)	Pilot multicenter RCT SpO2 of 88–92% vs. 96%in patients requiring MV of any cause (*n* = 103)	No difference in mortality or length of ICU stay	21% of the conservative group had COPD vs. only 10% of the liberal. Actual comparison was SpO2 of 93.4 vs. 97%	Larger trial needed
Schernthaner et al. (24)	Retrospective observational studies comparing arterial blood gases and mortality in pulmonary edema and heart failure (*n* = 475)	Increased mortality in patients with pneumonia with arterial PO2 of 150 vs. 117 mmHG (HR = 1.02; 95% CI: 1–1.4, *p* = 0.02)	Pneumonia related Nontrial data Potentially measuring true hyperoxemia effects.	Optimal PO2 calculated as 98 mmHg (or ~SpO2 of 97.3%)
van den Boom et al. (17)	Multicenter, retrospective observational analysis of SpO2 and mortality in ICU patients of any cause (*n* = 35,000)	Increased mortality with mean SpO2 92 vs. 96% [OR, 3.2 (2.9–3.5)]. Increased mortality with mean SpO2 100 vs. 96% [OR 1.6 (1.5–1.6)] (*n* = 26,723).	Large patient numbers utilizing shared datasets. Not a clinical trial.	Optimum target SpO2 is 94–98%
IICU-ROX Investigators the Australian New Zealand Intensive Care Society Clinical Trials Group et al. (20)	Multicenter, RCT SpO2 of 92–96% vs. normal in patients requiring MV of any cause (*n* = 1,000)	No difference in mortality or length of ICU stay	Actual Comparison was mean SpO2 of 95–96% vs. 96–97%	None made
Barrot et al. (21)	Multicenter, RCT SpO2 of 88–92% vs. >96%in patients with ARDS of any cause (*n* = 205)	Study halted due to safety. Significantly higher mortality in the conservative oxygen group. Ninety-day mortality was 44% in conservative arm vs. 30.4% in liberal arm.	Actual Comparison was mean SpO2 of 92–93% vs. 95–97%	None made

*RCT, randomized controlled trial; SpO2, oxygen saturations; HR, hazard ratio; RR, relative risk; OR, odds ratio (adjusted); ICU, intensive care unit; MV, mechanical ventilation*.

Hypoxia (SpO2 <95%) has numerous adverse health effects. In the acute setting, hypoxia increases the risk of fatal arrhythmia and end-organ damage (26). In a subacute setting, hypoxia has been shown to drive pulmonary inflammation and the systemic inflammatory response (27, 28) and to promote coagulation leading to an increase in thromboembolic events (29, 30). Long-term effects of hypoxia include ongoing cognitive impairment (31).

In relation to pneumonia specifically, delayed correction of hypoxia has been shown to lead to a more protracted and severe pneumonia, an increase in the rate of mechanical ventilation, and an increase in actual mortality (32, 33).

There is no evidence to suggest that the pathophysiology of COVID-19 pneumonia is exempt from these established detrimental effects of hypoxia. Indeed, there is evidence to suggest earlier correction of hypoxia in COVID-19 pneumonia may lead to improved outcomes. Shenoy et al. highlight the exacerbation of hypoxia on angiotensin-converting enzyme 2 (ACE2) upregulation and pulmonary vasoconstriction in COVID-19 pneumonia, suggesting that “permitted hypoxia” may be leading to more severe disease (34). Ackermann et al. examined autopsy specimens from influenza (H1N1) and COVID-19 cases and found a greater level of intussusceptive angiogenesis and alveolar microthrombi in COVID-19—both of which can be caused/exacerbated by hypoxia (35). It is of note that the H1N1 outbreak did not incur oxygen rationing.

Of the large postmortem cohort studies, an Italian cohort (*n* = 38) and a US cohort (*n* = 67) both report a hypercoaguable state with pulmonary thromboembolism in medium or small arteries in over 80% of COVID-19 lung specimens examined (36, 37). Both the US and Italy adopt a conservative oxygen strategy [SpO2 <92% prior to the commencement of oxygen (10, 11)]. The largest Swiss postmortem cohort (*n* = 21), where standards of oxygen targets have been maintained (SpO2 of 94–98%) (38), found that only 19% had evidence of peripheral or central pulmonary thromboembolism (39). There are inadequate numbers of postmortem studies globally to generate any form of conclusions, but given that hypoxia is known to cause a hypercoaguable state, likely to be exacerbated under proinflammatory conditions, and hypoxia itself further promotes pulmonary inflammation, we should be mindful of the likelihood that delayed correction of hypoxia in COVID-19 patients increase the propensity for, and therefore damage caused by, thromboembolism of the pulmonary vasculature.

In-keeping with this, Sun et al. reported a reduction in ICU admissions and mechanical ventilation by early identification and correction of hypoxia in patients with COVID-19 (40).

While such evidence is welcomed, we must be clear that it is not a requirement for the established pneumonia guidelines to prove efficacy in COVID-19 pneumonia. Target oxygen saturations in acute illness, pneumonia, and COVID-19 pneumonia remain at SpO2 of 94–98% (14, 25, 41). In the complete absence of any evidence to support an improved outcome—or even convincing evidence of the same outcomes—for lower target oxygen saturations in COVID-19 pneumonia, and with the more recent trials showing a convincing increase in mortality of conservative oxygen strategies, clinicians must continue to advocate for improved access to care and must not accept suboptimal or harmful amendments to established standards of care, certainly not based on an evidential argument.

While the scientific evidence provides no defense for lowering target oxygen saturations in COVID-19 pneumonia, there may be local civil contingency or procurement rationale for implementing such policies. The UK-wide directive to ration oxygen to patients, issued by the National Health Service (NHS) England in April 2020, may have been such a resource-related recommendation (7). Such a directive—where the evidence for harm is substantial—poses a considerable challenge to healthcare providers. The evidence is quite clear that a delay in the initiation of oxygen to the hypoxic, pneumonic patient leads to higher rates of mechanical ventilation, prolonged hospital stays, higher mortality, and, crucially, from a civil contingency standpoint, the real possibility of an overall increased consumption of oxygen supplies. There is also no evidence to suggest that COVID-19 pneumonia will differ in this regard. As such, and while we appreciate the challenging decisions relating to resource allocations, we must continue to advocate for improved access to treatment. That is, the decision to conserve oxygen and reduce target oxygen saturations in COVID-19 patients may well be made on a resource-limitation perspective, but it is done so within the fully transparent evidence that such compromise will cost lives.

The identification of the COVID-19 phenomenon of “silent hypoxia” adds further levels of complexity to frontline healthcare providers. The evidence already alluded to clearly support efforts to maintain the standards of care of COVID-19 patients to at least that afforded patients with other forms of viral pneumonia. Given that there are these “silent hypoxic” COVID-19 patients who do not complain of shortness of breath yet suffer marked hypoxia at rest, then—if the aim is to reduce mortality—we cannot rely on the self-reported symptom of breathlessness to identify those requiring further assessment and/or supplemental oxygen (42). Lower thresholds for measuring oxygen levels in patients suspected or confirmed to have COVID-19 may be prudent. This of course challenges the “stay home” approach adopted by a number of nations in favor of an “early assessment and ongoing vigilance” approach as adopted by the likes of Singapore, Australia, and Japan.

Similar to Middle East respiratory syndrome (MERS) and SARS, evidence is growing of a prolonged recovery time, and disability, following COVID-19 pneumonia (43). The largest study to date examined 548 patients more than 3 months after discharge from hospital and compared them to local controls. Half of the patients suffered prolonged symptoms (fatigue/physical decline, dyspnea, tachycardia, and alopecia), but <10% had persistence of these symptoms at 3 months. Of considerable note is that the only acute symptom present on initial admission to hospital that was associated with persistent symptoms (fatigue/physical decline, dyspnea, and tachycardia) was shortness of breath. A further UK cohort revealed a startling 85% of patients suffering prolonged symptoms who stayed home during the initial infection suffered breathlessness (*n* = 164) (38). Prolonged hypoxia during the active infection may account for some of the more prolonged disability following COVID-19.

Many countries have not taken a “conservative” oxygen approach to COVID-19. Most notably, Singapore, Switzerland, and Austria have maintained target SpO2 of >94% throughout the outbreak (24, 44); (45). Other countries with similar population burdens of total COVID-19 cases have taken a conservative oxygen approach. Most notably, the US, UK, Italy, France, and Spain have all maintained guidelines permitting a delay in the initiation of oxygen until oxygen saturations are ≤ 92% (8–12). Within the context of reduced vigilance and an overexuberant “stay home” message, the excess mortality caused by such conservative oxygen strategies are likely to be compounded further. Most certainly, patients with COVID-19 require more care and attention, not less.

The reason some nations have employed a “conservative oxygen strategy” remains largely unknown. The UK, and perhaps the US, was concerned about oxygen provisions (7, 10). The UK specifically was concerned with the “rate of flow” (not the actual oxygen supply). Would the system (pipes and valves) handle the increased draw? There is also the possibility that the implementation of a conservative oxygen strategy was motivated to permit more patients to be managed at home or indeed to remove them from healthcare follow-up altogether and, as such, relieve the healthcare system. No modeling regarding such an approach has been published. Indeed, failing to correct hypoxia earlier is likely to compound the pressures on the high-intensity, high-skilled clinical care areas (e.g., Respiratory and Intensive Care Wards).

## Conclusion

During a surge of cases that overwhelms a local healthcare system, there will be many compromises. It is likely that some of these compromises will have an impact on morbidity and mortality. It is then important to readjust quality and standards back to optimum when a healthcare system begins to recover or, where the healthcare system continues to struggle, place greater emphasis and effort into building healthcare capacity.

A conservative oxygen approach is a compromise that carries a significant mortality impact in COVID-19 pneumonia. Just as in other pneumonias, the time taken to correct hypoxia relates to disease severity, disease burden, and mortality. As the focus shifts to re-establishing healthcare capacity, improving the identification of the hypoxic patient and improving access to supplemental oxygen—delivered optimally—likely represent an appreciable modifiable factor in the bid to reduce the morbidity and mortality associated with the COVID-19 pandemic.

With regard to target oxygen saturations specifically, the evidence is clear: target oxygen saturations for the majority of people with COVID-19 remain at 94–98% [for acidotic type 2 respiratory failure see specific guidelines (25)].

## Data Availability Statement

The original contributions presented in the study are included in the article, further inquiries can be directed to the corresponding author/s.

## Author Contributions

DG, FM, and SB contributed to the conception and writing of the article.

## Conflict of Interest

The authors declare that the research was conducted in the absence of any commercial or financial relationships that could be construed as a potential conflict of interest.
